# Reports on the Hospitals of the United Kingdom

**Published:** 1910-06-18

**Authors:** Henry Burdett


### REPORTS
### ON THE
### Hospitals of the United Kingdom.

By SIR HENRY BURDETT, K.C.B., K.C.V.O.

### LI.—ROYAL ALEXANDRA HOSPITAL FOR SICK CHILDREN, BRIGHTON.

This institution was established in 1868 by the late Dr. Taffe, who had to fight a strenuous battle to get a footing for this special hospital, in which we had some hand at the time. It contains 57 beds with an average occupancy of 44.

This hospital is pleasantly situated; the wards face the large garden space, where there is an extensive verandah, provided by Mr. Arthur Wagg, upon which many a small child has recovered health and strength and been restored to a useful existence. We found the hospital well kept for a children's hospital, the children being particularly well cared for and appearing supremely happy. Every child knew the sisters well, and regarded them with evident affection and confidence. The exterior of the building is attractive, and the designing and grouping of the buildings is attractive too. We could wish that the architect had had some experience in hospital building, for then he would probably have spent less energy upon the façade, suitable as it is, and so have avoided the many noticeable defects, in the nurses' home especially, where light and air are excluded in more than one instance, in order to provide things which were apparently forgotten in the original plan, such as a box-room, slop sinks, and so forth. The actual bedrooms for nurses are good, but we would suggest that in place of the board-room the committee should furnish and equip the large workroom upstairs and so convert it into a nurses' sitting and recreation room.

This hospital, so far as the ground and first floor wards are concerned, is worthy of a visit in order to see how unwise it is for hospital authorities to adopt patent materials which have not been adequately tried and proved to be durable and suitable for hospital purposes. The walls of these two large wards have been treated by an Italian artist, who started out to apply some special preparation which would give the appearance of marble walls. These walls to-day are not attractive, and the sooner they are painted and put in order the better. The laundry does not contain the modern machinery with which it should be equipped, and we hope this may soon be supplied.

Operations are performed in the casualty department of the out-patient branch, but cases of adenoids are taken in after operation for one night. This plan is not generally followed by hospitals nowadays, but it has much to commend it. We, however, hope that some generous donors will follow the example of Mr. Arthur Wagg and provide similar verandah accommodation for the first and second floor wards, so that it may be possible to enable suitable cases to sleep out in the open air, an arrangement which the experience of the ground floor verandah shows to be highly beneficial to many children.

We were pleased to find Miss Fraser, who was trained at the Seamen's Hospital, occupying the post of matron. She is evidently devoted to the interests of the institution, and is doing good work with zeal and ability.

**Some Smaller Hospitals** will be reported on next week.

